# Influenza Vaccine Effectiveness among US Military Basic Trainees, 2005–06 Season

**DOI:** 10.3201/eid1304.061308

**Published:** 2007-04

**Authors:** Jennifer K. Strickler, Anthony W. Hawksworth, Christopher Myers, Marina Irvine, Margaret A.K. Ryan, Kevin L. Russell

**Affiliations:** * Naval Health Research Center, San Diego, California, USA

**Keywords:** human influenza, vaccine effectiveness, military personnel, dispatch

## Abstract

Virtually all US military basic trainees receive seasonal influenza vaccine. Surveillance data collected from December 2005 through March 2006 were evaluated to estimate effectiveness of the influenza vaccine at 6 US military basic training centers. Vaccine effectiveness against laboratory-confirmed influenza was 92% (95% confidence interval 85%–96%).

Public health concerns over the potential for a devastating influenza pandemic in the near future are well known. Surveillance efforts have increased throughout the world, and much time and money have been directed toward preparedness for such a pandemic. Given that vaccination rates vary greatly among the nonmilitary population and that influenza diagnostics are sporadically available, annual influenza vaccine effectiveness studies based on laboratory-confirmed diagnoses are rare. However, evidence of locally circulating strains evading the vaccine-induced protection could be critical for early recognition and intervention. In addition, the emergence of pandemic strains within military populations has been noted. The first documented influenza outbreak in the spring of 1918, before the great influenza pandemic of 1918–19, was among recruits at Fort Riley, Kansas (*1*). In 1976, a unique strain of influenza (H1N1) caused an outbreak at Fort Dix, New Jersey, causing 1 death, and creating concern over spread of this nonvaccine strain (*2*). Highly vaccinated military populations, under close surveillance, provide the opportunity for annual calculation of influenza vaccine effectiveness, thereby benefiting global pandemic preparedness.

## The Study

The Naval Health Research Center (NHRC) began conducting tri-service surveillance for febrile respiratory illness at military training centers in 1996; by 1999, this surveillance network had expanded to include 8 of the largest military basic training centers in the United States (*3*). This surveillance includes the systematic collection of throat swab specimens and clinical data (including but not limited to gender, date of birth, symptoms, influenza vaccination status, type of vaccine received, and date of vaccination) from consenting US military trainees meeting the case definition for febrile respiratory illness (oral temperature ≥100.5°F [38.0°C] and a cough or sore throat). Samples are stored locally at each site at −70°C until they are forwarded to the Naval Respiratory Disease Laboratory at NHRC for viral culture and molecular diagnostic processing. Research personnel at participating surveillance sites report the weekly number of trainees who sought care for febrile respiratory illness and total trainee populations for their respective sites, and rates for such illnesses are calculated.

During the 2003–04 influenza season, we recognized the opportunity of using data from this ongoing active surveillance to estimate influenza vaccine effectiveness in protecting against both laboratory-confirmed influenza and febrile respiratory illness of any cause among US military basic trainees. Despite concerns that vaccine effectiveness during the 2003–04 season would be low because of the poor match between the components of the vaccine and the circulating strain (*4*), the vaccine provided good protection (94.4%) against laboratory-confirmed influenza that season (*5*). Annual vaccine effectiveness calculations are important as we heighten our preparedness for pandemic influenza strains; therefore, we performed similar calculations for the 2004–05 and 2005–06 seasons.

During the late fall and winter seasons, all active-duty military forces are required to receive the influenza vaccine, and this policy is strictly enforced in training camps. Upon arrival, all incoming trainees receive mandatory influenza vaccination, either the trivalent inactivated influenza vaccine by injection (FluZone, Sanofi Pasteur, Lyon, France) or intranasal cold-adapted, live, attenuated influenza vaccine (CA-LAIV) spray (FluMist, MedImmune, Gaithersburg, MD, USA).

For this analysis, vaccine protection was assumed to begin 14 days postvaccination. Therefore, in an 8-week training program, 25% of trainees were considered “unvaccinated” at any given time, assuming immunity takes 14 days to develop. Likewise, 33% of trainees in a 6-week training program were considered unprotected by the vaccine at any time. These assumptions allow estimates of denominator data for “vaccinated” and “unvaccinated” person-weeks in calculations of vaccine effectiveness..

From January through March 2006 all new trainees arriving for basic training received the influenza vaccine; all recruits already present had been vaccinated. The observation period for this analysis included January 1—March 31, 2006. However, 2 sites, Naval Service Training Command, Great Lakes, and Marine Corps Recruit Depot, San Diego, had completed vaccination by December 2005. Therefore, December was included in the observation period for those sites as well. Total person-weeks in recruit training during the observation period were obtained directly from the participating training centers. Vaccine effectiveness was calculated for both laboratory-confirmed influenza and any cause of febrile respiratory illness as follows: 100 × (1 – relative risk = 1 – [rate in vaccinated group]/[rate in unvaccinated group]).

During the observation period, 6 of 8 surveillance sites had influenza activity and were included in this analysis. In 479,181 person-weeks of observation, 4,052 cases of febrile respiratory illness were reported from these 6 sites, and 722 patients were enrolled into the surveillance study (includes throat swab specimen, case data, and consent). Seventy (9.7%) specimens tested positive for influenza, by either culture or molecular techniques.

Rates of laboratory-confirmed influenza were higher among unvaccinated trainees at all sites except Fort Benning, Georgia, which had only 3 cases (Figure). Overall, influenza vaccine effectiveness among US military trainees was 92% (confidence interval [CI] 85.4–95.6%) during the 2005–06 season (Table). Vaccine effectiveness against laboratory-confirmed influenza was high (range 86%–94%) in each of the past 3 seasons. Vaccine effectiveness against non–laboratory-confirmed febrile respiratory illness was lower, ranging from −10% in 2005–06 to 52% in 2004–05.

**Figure:**
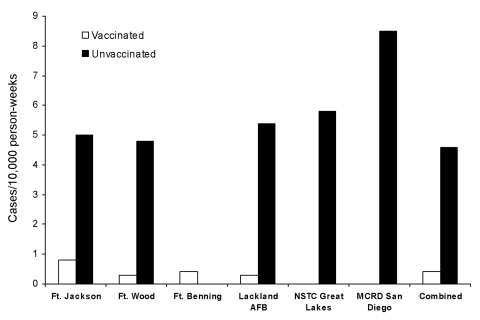
Incidence of laboratory-confirmed influenza by vaccination status. AFB, Air Force base; NSTC, Naval Service Training Command; MCRD, Marine Corps Recruit Depot.

**Table:** Vaccine effectiveness against laboratory-confirmed influenza among US military basic trainees, 2005–06*†

Site	Vaccinated person-weeks	Unvaccinated person-weeks	Cases in vaccinated trainees	Cases in unvaccinated trainees	Vaccine effectiveness (%)	95% CI
Fort Jackson, SC	77,874	25,958	7	13	82.1	
Fort Wood, MO	67,513	22,504	2	11	93.9	
Fort Benning, GA	68,652	22,884	3	0	–	
Lackland AFB, TX	37,435	18,690	1	10	95.0	
NSTC Great Lakes, IL	67,763	22,588	0	13	100.0	
MCRD San Diego, CA	35,490	11,830	0	10	100.0	
Total	354,727	124,454	13	57	92.0	(85.4%, 95.6%)

*CI, confidence interval; SC, South Carolina; MO, Missouri; GA, Georgia; AFB, Air Force base; TX, Texas; NSTC, Naval Service Training Command; IL, Illinois; MCRD, Marine Corps Recruit Depot; CA, California. †Assuming 14 d before vaccine is protective.

## Conclusions

This analysis suggests that the 2005–06 influenza vaccine was highly effective in protecting US military basic trainees against laboratory-confirmed influenza. Furthermore, these data suggest that both the trivalent inactivated vaccine injection and the CA-LAIV intranasal spray were equally effective, because the Marine Corps Recruit Depot in San Diego vaccinated its trainees with CA-LAIV almost exclusively, and vaccine effectiveness at that site was 95% (vaccine effectiveness at all other sites combined = 90%).

These estimates of effectiveness were supported by results of additional analyses that would be expected to bias the outcome toward the null hypothesis. For example, a 7-day lag period before immune response was considered in an alternative analysis, and it yielded similar results: the calculated vaccine effectiveness changed only slightly, from 92% to 90%. We also analyzed vaccine effectiveness, assuming that 10% fewer trainees were vaccinated at any given point, yet the calculated vaccine effectiveness was only reduced to 87%.

In contrast to the consistently high effectiveness of the vaccines against laboratory-confirmed influenza, the effectiveness against febrile respiratory illness of any cause was much lower and varied with each season (13.9% in 2003–04, 52.1% in 2004–05, and −10% in 2005–06). This lower effectiveness in 2005–06 is most likely due to the generally high proportion of adenovirus infection seen in this population (*6*), and the lesser effectiveness is further exacerbated by the tendency for adenoviral infections to occur beyond the second week of training. The lower vaccine effectiveness seen against febrile respiratory illness of any cause gives credence to the estimates of high vaccine effectiveness against laboratory-confirmed influenza. If a measurement bias existed, both estimates would be affected.

As a highly vaccinated population, military personnel, and basic trainees in particular, can provide critical information regarding the effectiveness of each year’s influenza vaccine formulations. Because of the annual variations of both the vaccine formulations and the circulating strains, influenza vaccine effectiveness should be evaluated annually. With the ever-rising concerns of an imminent influenza pandemic, reliable and rigorous influenza surveillance is paramount. Our existing surveillance network will allow us to repeat the methods used in this analysis each year, thus providing valuable estimates of influenza vaccine effectiveness to the public health community.
